# Endocrine, energy, and lipid status during parturition and early lactation in indigenous goats native to the Algerian Sahara

**DOI:** 10.14202/vetworld.2021.2419-2426

**Published:** 2021-09-18

**Authors:** Kamilia Henna, Sofiane Boudjellaba, Farida Khammar, Zaina Amirat, Didier Chesneau, Salima Charallah

**Affiliations:** 1 Department of Biology and Physiology of Organisms, University of Science and Technology Houari Boumediene, Faculty of Biological Sciences, Laboratory of Research on Arid Lands, BP 32 El Alia, 16111, Algiers, Algeria; 2 Department of Pre-Clinic, Higher National Veterinary School, Laboratory of Research Management of Local Animal Resources, Abbes Street, Oued-Smar, 16000, Algiers, Algeria; 3 Department of Animal Physiology and Farming System, University of François Rabelais, F-37041, Tours, Reproductive and Behavioral Physiology, National Institute of Agriculture, Food and Environment, INRAE UMR85, ER 11, Neuroendocrinology of sexual interactions and behaviors, CNRS, IFCE, Nouzilly, France.

**Keywords:** hormones, lactating goat, metabolic profile, parturition, Sahara

## Abstract

**Background and Aim:**

Goats are widely distributed in southwest Algeria. The Saharan goat is perfectly adapted to the harsh conditions of arid areas, and it is characterized by resistance to long photoperiod and reduced metabolic needs, allowing the survival of its offspring by maintaining lactation. Several studies have demonstrated that parturition and lactation are critical periods that induce hormone, energy, and lipid status changes in mammals. However, the relationship between the blood biochemical parameters of parturition control and lactation functions in the Algerian Saharan goat has not been thoroughly documented. Therefore, this study assesses hormone and metabolite levels during parturition and early lactation in Saharan goats reared in arid areas.

**Materials and Methods:**

Experiments were performed on 14 multiparous female goats, and blood samples were collected during parturition, 4 days postpartum (D1PP-D4PP), and during the first 12 weeks of lactation (W1-W12) to analyze prolactin, cortisol, glucose (GLU), total proteins (TP), cholesterol (CHO), triglycerides (TGs), total lipids (TL), low-density lipoproteins (LDLs), high-density lipoproteins (HDLs), and very LDLs (VLDLs).

**Results:**

Statistical data analysis revealed a significant (p<0.05) increase in plasma prolactin concentrations at W1 after parturition, reaching maximum values at W3 and W9, and remained high until W12 of lactation. Plasma cortisol levels were high at parturition, reaching two peaks at W3 and W9, and then decreased at W5, W7, and W12 of lactation. No significant changes were found in serum GLU levels during the first 7 weeks of lactation compared with parturition day; then, the levels became significantly (p<0.05) lower at W8, W11, and W12 of lactation. Plasma TP increased significantly (p<0.05) at D3PP, W1, and W4, then decreased significantly (p<0.05) at W8. In addition, this decrease coincided with that of GLU production. Serum CHO, TGs, TL, LDLs, and VLDLs, were low at parturition and high at D4PP and during the first 3 months of lactation. Furthermore, HDL levels were low at D3PP, 1^st^, and 3^rd^ months and high at the 2^nd^ month of lactation.

**Conclusion:**

This study emphasized the impact of parturition and the 1^st^ weeks of lactation on endocrine and metabolic changes in indigenous goats living in the Algerian Sahara Desert. These results can be used to monitor and improve farming management and understand physiological adaptive strategies, mainly lactation function sustainability, of this goat living in marginal zones.

## Introduction

Indigenous goats play a crucial role in arid agricultural zones where the Saharan populations depend on them for livelihood. Several ecophysiological and metabolic studies have demonstrated their adaptive physiological characteristics to arid areas. Indeed, in these animals, the reduction of water turnover and glomerular filtration rates is observed [1], and high levels of placental pregnancy-associated glycoproteins are produced, which maintain gestation [2].

Our laboratory has conducted several studies on this local breed, focusing on its reproductive endocrine ecophysiology and pathophysiology [2-6] and endocrine and metabolic response in the Saharan environment [7,8]. Recently, the performance of lactating goats based on relationships between milk yield and udder morphological traits was investigated [9]. The low milk production has been demonstrated, since the average daily milk yield was 0.56 kg, reaching its peak (0.71 kg/day) at week 6 of lactation. This is related to the mammary gland conformation adapted for grazing in desert ranges but not suitable for high production; however, it does not prevent lactation sustainability and growth of kids. Therefore, assessing the endocrine and metabolic status during lactation is essential to better understand how this breed maintains lactation in an arid environment. Moreover, the mammary gland is a target of several hormones, such as prolactin and cortisol, which act synergistically to upgrade its development and function. Prolactin involved in mammary gland development controls the secretion and sustainability of milk by activating the gene transcription of caseins and enzymes and endoplasmic reticulum development. In addition, it increases neurogenesis and insulin resistance [10], while cortisol is essential for secretory activation, ample milk synthesis [11], maintaining homeostasis, and animal adaptation [12]. Furthermore, blood metabolic profile (BMP) is used to predict the emergence of some metabolic disorders, energy metabolism, and nutritional and health status in animals [13]. The indicators of BMP are mainly the hematochemical parameters, which are influenced by various factors, such as breed, age [14], gender [15], season [16], reproductive status [17], and infectious processes [18]. The most important biomarkers of energy metabolism are glucose (GLU), cholesterol (CHO), and triglycerides (TGs). The relationship between BMP and lactation function control has been previously reported in several healthy goats of various breeds [17,19,20]. However, there is a lack of information concerning the data of hematochemical parameters during parturition and lactation period in the Saharan breed.

This study aimed to describe the physiological changes in hormone and metabolite parameter concentrations during these reproductive phases in goats native to the Algerian Sahara Desert.

## Materials and Methods

### Ethical approval

The animal experiment was approved by the Ethical Committee of the Algerian Higher Education and Scientific Research (Executive Decrees No. 04-82 and No. 10-90) and agreed by the Algerian Association of Sciences in Animal Experimentation (AASEA, agreement number 45/DGLPAG/DVA.SDA.14) of the University of Science and Technology Houari Boumediene, of Algiers.

### Study period and location

The study was conducted from October 2014 to September 2018 in Southwest Algeria.

### Animals and management

Fourteen healthy multiparous goats (*Capra hircus*), 2-8 years old, weighing 19.6±4.03 kg, were used. Animals were kept in the sheepfold of the Béni-Abbès experimental station located in the Algerian Sahara Desert (30°07′N., 2°10′W.; elevation 497 m). The climate of this region is classified among the hottest and driest in Algeria, where ambient temperature can reach 47°C in the summer. The annual rainfall recorded is 18 mm in autumn and winter. During the breeding season (autumn), the females were naturally mated with a fertile buck; in the spring, all pregnant goats gave birth to 16 kids, including seven males and nine females. Twelve dams give birth to single kids and two gave birth to twins; among them, 13 dams did not present reproductive disorders, such as dystocia, retained placenta, and uterine infections. However, the 14^th^ dam gave one kid from twin delivery, which died at birth due to dystocia. The dams were also healthy from any mammary gland infections (mastitis and udder edema) and incidences of metabolic disorders, such as milk fever, displacement of the abomasum, fatty liver syndrome, and ketosis. The kids permanently stayed with their mothers and were weaned at the age of 3 months. The dams were fed twice daily with a ration of 0.6 kg/goat of forage cereals and 0.6 kg/goat of barley supplemented with dates and *Aristida pungens* (known in Arabic as “drinn”) throughout the experimental period and green alfalfa during the first postpartum (PP) days. Water and stones to lick were available *ad libitum*.

### Blood sampling

Blood samples were collected from the external jugular vein at 08:00 a.m. before feeding. All animals were sampled at parturition (D0), PP days (D1PP-D4PP), and weekly for 12 weeks of lactation (W1-W12). The blood samples were stored in two vacutainer tubes; one contained lithium heparin for hormones and total protein (TP) assays, and the other had no anticoagulant for serum metabolites analysis. The samples were centrifuged at 3000×g for 15 min at 4°C. The decanted sera and plasma were stored in Eppendorf microtubes at −20°C until analysis.

### Hormone assays

#### Prolactin radioimmunoassay (PRL-RIA)

Plasma PRL was assayed in duplicate using a heterologous double-antibody RIA according to Kann [21] and Orgeur *et al*. [22]. The antibody anti-PRL obtained in rabbits was provided by the INRA-PRC laboratory (Nouzilly, France), and the anti-rabbit antibody (SMAL) was obtained in the ovine. The radioactivity fraction of the precipitate was quantified using a gamma counter (Packard, PerkinElmer, USA). Intra- and inter-assay coefficients of variation were 8% and 13%, respectively. Assay sensitivity was 2.5 ng/mL.

#### Cortisol radioimmunoassay (CORT-RIA)

Plasma cortisol was analyzed in duplicate, according to Murphy [23]. Cortisol antisera were produced in rabbits (Sigma-Aldrich, C8409, USA). The tritium-labeled cortisol ([1,2,6,7-^3^H (N)], PerkinElmer, NET396) was obtained from CEBC-CNRS, France. The radioactivity of the precipitate was quantified using a Beta counter (Tri-Carb 2810 TR, Liquid Scintillation Analyzer, PerkinElmer). Intra- and inter-assay coefficients of variation were 14.4% and 16.4%, respectively. The sensitivity of the assay was 0.16 ng/mL.

#### Biochemical assays

The serum metabolites were analyzed using an automated clinical chemistry analyzer (Bio lis 24i Premium Tokyo Boeki Medisys Inc. Japan) and a spectrophotometer. Bioassay kits (Biomaghreb, Tunisia) were used to assess GLU, CHO, and TGs, while Spinreact kits (S.A/S.A.U. Ctra. Santa Coloma, Spain) were used for high-density lipoprotein (HDL) analysis. Plasma TPs were measured using an auto-analyzer (Pentra C200. Horiba, France) and a commercial kit (ABX Pentra TP CP, France). Enzymatic colorimetric methods were used to assess serum GLU (GLU-oxidase/peroxidase), serum CHO and HDL (CHO oxidase/peroxidase), serum TG (glycerol 3-P-oxidase/peroxidase), and plasma TP (Biuret method). All coefficients of variation for intra- and inter-assay were <10%, ranging from 0.22 to 8.88% and 1.70 to 6.34%, respectively. Low-density lipoproteins (LDL) and very LDL (VLDL) levels were calculated according to Friedewald *et al*. [24]. Total lipid (TL) levels were estimated using the formula TL=(CHO×2.56)+(TG×0.87).

### Statistical analysis

The data from different variables were expressed as means±standard error of the mean (SEM) using SPSS for Windows v.20.0 (IBM Corp., NY, USA). The differences between various ages were estimated using Student’s t-test for paired samples and normally distributed data. Wilcoxon test was used for the data with normality lower than p<0.05. p<0.05 was considered statistically significant.

## Results

### Plasma prolactin and cortisol concentrations

**Figure-1:**
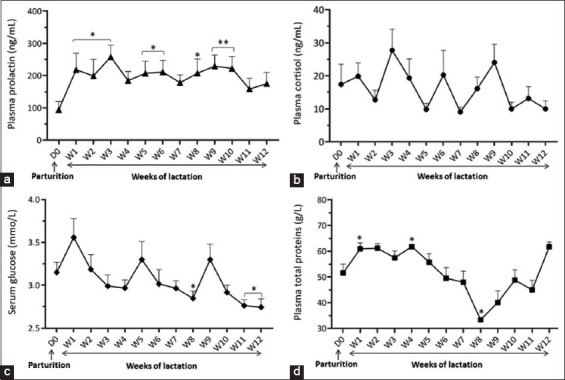
Circulating profiles of prolactin (a), cortisol (b), glucose, (c) and total proteins (d) at parturition (D0) and early lactation (W1 to W12) in indigenous goats reared in Algerian Sahara. (Mean±SEM, n=14). * indicate p<0.05, ** indicate p<0.01.

Our data showed non-significant (p>0.05) differences in plasma PRL and CORT concentrations from parturition (D0) to 4 days PP (Table-1) [25-30]. Plasma PRL levels ranged from 90.92 to 258.15 ng/mL and increased significantly (p<0.05) to reach a high level at W3 compared with parturition day (258.15±36.35 ng/mL *vs*. 96.49±28.21 ng/mL, respectively). The PRL concentrations remained high throughout the 12 weeks of lactation with a significant (p<0.01) increase at W9 and W10 of lactation (Figure-1a). CORT levels ranged from 9 to 26.76 ng/mL; however, no significant differences were observed during the experimental period. At parturition, the mean value was 17.40±6.12 ng/mL, and it decreased non-significantly at W2 then increased till a peak was recorded at W3 and W9 (26.76±5.98 ng/mL and 22.34±5.29 ng/mL, respectively), followed by a decline until W12 of lactation (9.50±2.22 ng/mL) (Figure-1b).

**Table 1:** Hematochemical parameters during parturition and postpartum days in indigenous goats reared in Algerian Sahara (mean±SEM, n=14).

Parameters	Parturition	Days postpartum				Reference values
	
	D0	D1PP	D2PP	D3PP	D4PP	
PRL (ng/mL)	94,63±24.94^a^	138,17±51.83^a^	90,92±20.73^a^	130,73±41.58^a^	130,79±41.17^a^	0-100 [25]
CORT (ng/mL)	17,40±6.12^a^	9,44±1.69^a^	13,34±4.06^a^	15,55±3.52^a^	11,36±2.23^a^	1-34 [26]
GLU (mmol/L)	3,14±0.11^a^	3,15±0.07^a^	3,26±0.11^a^	3,05±0.12^a^	3,30±0.10^a^	2.8-4.2 [27]
CHO (mmol/L)	1,28±0.06^a,d^	1,34±0.08^a,c^	1,46±0.06^b,c,e^	1,53±0.13^d,e,g^	1,64±0.05^f,g^	1.07±0.13 [28]
TG (mmol/L)	0,11±0.02^a,d^	0,12±0.01^a,c,d^	0,13±0.02^a,e^	0,16±0.03^d,f^	0,20±0.03^b,c,e,f^	0.16-1.6 [29]
TP (g/L)	51,54±3.44^a,c^	59,84±1.54^a^	42,10±4.44^c,d^	65,64±1.86^b^	55,36±3.80^a,d^	63-85 [29]
TL (g/L)	1,32±0.06^a,d^	1,42±0.08^a,c^	1,54±0.07^b,c^	1,64±0.15^d,f^	1,77±0.05^e,f^	NA
LDL (mmol/L)	0,13±0.04^a,d^	0,24±0.05^a,c^	0,33±0.06^b,c,e^	0,44±0.11^d,e,g^	0,49±0.05^f,g^	0.72±0.05 [30]
HDL (mmol/L)	1,11±0.06^a^	1,04±0.04^a^	1,07±0.03^a^	1,02±0.04^a^	1,06±0.04^a^	1.68±0.11 [30]
VLDL (mmol/L)	0,05±0.01^a,b^	0,05±0.01^a,b^	0,06±0.01^a^	0,07±0.02b,^c^	0,09±0.01^a,c^	NA

Wilcoxon test was applied for all hematochemical parameters. Means in the same line with different superscript letters are significantly different (p<0.05). Values with the same letter did not differ significantly. D0=Parturition, D1-D4=Postpartum days, NA=Not available. PRL=Prolactin, CORT=Cortisol, GLU=Glucose, CHO=Cholesterol, TG=Triglycerides, TL=Total lipids, TP=Total proteins, LDL=low-density lipoprotein, HDL=High-density lipoprotein, VLDL=Very low-density lipoprotein

### Concentration of biochemical parameters

The obtained results revealed that serum GLU levels ranged from 2.73 to 3.54 mmol/L and gradually increased (p>0.05) with slight fluctuations from parturition to D4PP (Table-1). The highest glycemic levels were recorded on W1 of lactation, which declined until W7; this decrease became significant (p<0.05) at W8, W11, and W12 of lactation (Figure-1c).

Plasma TP ranged from 33.33 to 65.64 ɡ/L and increased progressively from D0 to D1PP, peaking (p<0.05) at D3PP with a mean value of 65.64±1.86 g/L (Table-1). The plasma TP concentration increased significantly (p<0.05) at W1 and W4 of lactation, which gradually decreased until W8 compared with parturition (33.33±0.51 g/L *vs*. 51.54±3.44 g/L; p<0.05, respectively). A gradual increase followed this decrease until W12 of lactation (Figure-1d).

Serum CHO ranged from 1.28 to 1.64 mmol/L; significant (p<0.05) high concentrations of CHO were obtained at D4PP (Table-1), 1^st^, and 2^nd^ months of lactation compared with parturition (Table-2). Serum TG ranged from 0.11 to 0.20 mmol/L and increased (p>0.05) gradually from D0 to D2PP (Table-1); this increase became significant, reaching a maximum level at D3PP and D4PP (0.16±0.03 mmol/L and 0.20±0.03 mmol/L, respectively; p<0.05) compared with the time of parturition (0.11±0.02 mmol/L). TG levels increased non-significantly during the 3 months of lactation (Table-2). Serum TL ranged from 1.32 to 1.80 ɡ/L and significantly increased (p<0.05) progressively from D0 to D4PP recorded at D3PP and D4PP (Table-1); this rise remained until the 3^rd^ month of lactation compared with the day of parturition (Table-2).

**Table 2:** Serum lipids during parturition and early lactation stages in indigenous goats reared in Algerian Sahara (mean±SEM, n=14).

Parameters	Parturition	First months of lactation		
	
	D0	W1-W4	W5-W8	W9-W12
CHO (mmol/L)	1,28±0.18^a^	1.55±0.04^b,c^	1.63±0.05^b^	1.53±0.04^a,c^
TG (mmol/L)	0,11±0.02^a^	0.14±0.01^a^	0.16±0.01^a^	0.15±0.01^a^
TL (g/L)	1,32±0.05^a^	1.65±0.04^b,d^	1.80±0.05^c^	1.63±0.04^a,d^
LDL (mmol/L)	0,13±0.04^a^	0.42±0.03^b^	0.42±0.03^b^	0.45±0.04^b^
HDL (mmol/L)	1,11±0.06^a,b^	1.08±0.02^a^	1.15±0.02^a^	1.04±0.02^b^
VLDL (mmol/L)	0,05±0.01^a^	0.07±0.00^a^	0.07±0.00^a^	0.07±0.00^a^

Differences among the mean concentrations of each lipid parameter in the different groups were analyzed by Test t of Student. Mean in the same line with different superscript letters are significantly different (p<0.05). Values with the same letter did not differ significantly. D0=parturition, W1-W4=1^st^ month of lactation, W5-W8=2^nd^ month of lactation, W9-W12=3^rd^ month of lactation. CHO=Cholesterol, TG=Triglycerides, TL=Total lipids, LDL=Low-density lipoprotein, HDL=High-density lipoprotein, VLDL=Very low-density lipoprotein

Concerning the lipoprotein levels, serum LDL ranged from 0.13 to 0.49 mmol/L, increased significantly (p<0.05) from D2PP to D4PP (Table-1), and remained high until the 3^rd^ month of lactation (0.45±0.04 mmol/L) compared with the day of parturition (0.13±0.04 mmol/L) (Table-2). Serum HDL ranged from 0.99 to 1.19 mmol/L and decreased progressively (p>0.05) from D0 to D4PP (Table-1), then decreased gradually to reach a lower rate at the 3^rd^ month of lactation (Table-2). Finally, serum VLDL ranged from 0.05 to 0.09 mmol/L and increased progressively (p>0.05) from parturition to D4PP (Table-1), then fluctuated until the 3^rd^ month of lactation (Table-2).

## Discussion

This study provides significant variations in the endocrine and metabolic patterns during parturition and lactation in the indigenous goat, which is perfectly adapted to the adverse conditions of arid areas of Algeria. The hematochemical parameters investigated in this breed were in the physiological range (Table-1). PRL plays a fundamental role in the lactation of farm animals; it is the lactogenic hormone in mammals, which plays an important role in milk production control in dairy ruminants.

This study reported that plasma PRL increased with increased lactation with a peak at W3 and W9 of lactation. Similar results were reported in Turkish Saanen goat [31], whereas Castro *et al*. [32] reported that prolactinemia peaked at parturition and significantly dropped on the 1^st^ day of PP, followed by a sharp increase on the 4^th^ day after parturition. Moreover, in Israeli dairy Saanen goat, PRL level was higher at parturition then decreased progressively from W1 and W9 of PP [33]. In ruminants, the increase in prolactinemia after parturition is necessary for maintaining lactation [34]. This has also been confirmed in the Saharan breed, where we recorded elevated PRL levels throughout lactation. This rise could be due to the stimulation of suckling by kids; thus, inducing PRL release [35]. In addition, kids were kept with their dams and suckled *ad libitum* throughout lactation; this agrees with the elevated prolactinemia noted during the lactation period. It can also be related to a rise in ambient temperature, which can reach 47°C during summer. Furthermore, Sano *et al*. [25] found a strong positive correlation between PRL secretion and the increase in ambient temperature. Hence, high temperature is an exogenous factor stimulating the secretion of PRL, allowing the sustainability of lactation in arid areas. In contrast, low PRL concentration observed at parturition may be due to the increase in oestradiol-17b level, which inhibits the increase in PRL receptors in the mammary cells during pregnancy [36]. Furthermore, the decline in progesterone (P4) level at parturition induces a change in the P4/PRL ratio and an increase in the number of PRL receptors in the mammary gland. Furthermore, our results demonstrated two synergic high plasma PRL and CORT rates at W3 and W9 of lactation. Thus, CORT may be essential for growth and histological differentiation of the mammary gland and constitutes an enhancer of lactogenic hormone complex, particularly PRL. Plasma cortisol levels fluctuated from D0 to W12 with a high level at W3 and W9 of lactation. This result is similar to Turkish Saanen goat [31]. High cortisol levels are required to keep up intense milk synthesis and secretion during lactation; high cortisol levels recorded at parturition can be associated with labor stress, high ambient temperature, or feed restriction, which are severe stressors stimulating the hypothalamic-pituitary-adrenal (HPA) axis. According to the literature, several studies on lactating goats reared under arid and semi-arid climates reported that cortisol concentration does not exceed 9 ng/mL [16,37,38]. In contrast, we reported a higher cortisol level in our breed, reaching a value of about 26.76 ng/mL. This rise may be related to the ecophysiological responses to the arid environment, involving the activation of the HPA axis with an increase in corticotropin-releasing, antidiuretic, and adrenocorticotropic hormone production [8]. Consequently, the increase in CORT production promotes protein catabolism, and converting proteins into amino acids to support gluconeogenesis.

GLU is an essential energetic substrate and is an important component of lactose, which plays a primary role in milk production. The GLU levels obtained in the Saharan breed increased progressively from D0 to D4PP and peaked at W1 after parturition, then significantly dropped at W8, W11, and W12 of lactation. This decrease may be linked to high energy demands of the mammary gland for lactose milk synthesis, greater insulinemia activity, or an increase in ambient temperature, which negatively affects the hypothalamic center (appetite control), causing a low feed intake [39]. This reduction may also be associated with a negative energy balance, leading to lipolysis, which increases non-esterified fatty acid (NEFA) levels used for TG synthesis. This case supports our study results, which recorded a high triglyceridemia with low glycemia. Our results agree with those found in Brazilian dairy [40] and Croatian Alpine goats [41]. Hyperglycemia observed at W1 of lactation could be ascribed to the low responsiveness of peripheral tissues to insulin. In addition, an increase in milk production demonstrated that animals were in a positive energy balance and a high CORT level that increases hepatic gluconeogenesis; this case confirms our study where we recorded simultaneously high cortisol and GLU levels.

The low TP levels recorded at parturition in our study may be an adaptive response to the high requirement for water mobilization from blood to mammary glands for lactogenesis. Increased TP levels recorded during lactation are due to immunoglobulin transfer from the bloodstream to the mammary gland. Our study observed a significant decline in TP at W8, coinciding with that of GLU; thus, it allows the udder to increase lactose and immunoglobulin rates for milk production [42]. A remarkable decrease in TP was recorded from W5 to W9 of lactation, coinciding with a significant increase in PRL rates; this was explained by TP infiltration of the blood toward anterior pituitary lactotroph cells for synthesizing PRL, which acts directly on alveolar epithelial cells to increase the expression of genes for milk proteins and stimulates lipid and carbohydrate synthesis and the transport of ions in milk. This effect is potentiated by CORT, insulin, insulin-like growth factor, and growth hormone, which multiply the intracellular organelles essential for proteinogenesis.

CHO belongs to the sterol family and plays a key role in many biochemical processes. CHO and TG levels are used as an indicator of lipid profile. Cholesterolemia obtained was lower on D0, then peaked at D4PP and remained high until the 3^rd^ month of lactation. Our results agree with those of Aardi goats in Saudi Arabia [43] and red Syrian goats [44]. However, Iriadam [45] had a significant increase in parturition in Kilis goats. In the Saharan breed, hypocholesterolemia recorded at parturition can be due to adrenal and ovarian steroid production, fat-soluble vitamin production, and high thyroid hormone levels [46]. The latter inhibits the activity of b-hydroxy b-methylglutaryl CoA reductase, a crucial enzyme regulating CHO synthesis, whereas glucagon and glucocorticoids decrease it [40]. The hypercholesterolemia recorded during lactation suggests lipid mobilization mediated by glucagon, an acute synthesis of plasma lipoproteins, an important feed intake for milk synthesis, or the estrogens that stimulate CHO synthesis. This rise can also be due to a reduction in lipogenesis, lipid esterification, and catecholamine increase, which induces NEFA release [47].

TGs are major components of VLDL and chylomicrons and are considered an energy source and play a role in dietary fat transport. TG levels obtained increased from D0 until the 3^rd^ month of lactation. These data are similar to those reported by Allaoua and Mahdi [48] in Arbia goats. Hypotriglyceridemia recorded at D0, D1PP, and D2PP may be explained by milk fat production using peripheral blood of TG, hyperactivity of lipoprotein lipases, and NEFA level reduction [49]. However, hypertriglyceridemia observed during lactation may be related to hormonal regulation (P4, glucocorticosteroids, catecholamines, and glucagon) and NEFA level increase used for TG synthesis. Serum TL obtained in Saharan goats were low at D0 and high at D4PP until the 3^rd^ month of lactation. This rise can be related to the inhibition of apoprotein synthesis and their receptors, which are essential for VLDL and P4 production [50].

Concerning lipoprotein levels, serum LDL increased significantly from D4PP until the 3^rd^ month of lactation compared with parturition. Similar results were found in the Croatian Alpine [41] and the Maltese goats [47]; Tharwat *et al*. [51] reported higher LDL levels at parturition in Saudi Arabian goats. The high levels of LDL and CHO obtained in our breed reflect high cortisol levels during lactation, indicating the role of LDL in transporting CHO from plasma to cells of the adrenal cortex for CORT synthesis. Serum HDL showed a decrease at D3PP, 1^st^, and 3^rd^ months and increased at the 2^nd^ month of lactation. This is similar to that observed in other goat breeds [47,51,52]. Concerning serum VLDL, it increased from parturition until the 3^rd^ month of lactation. In addition, NEFA levels can be re-esterified in the liver mitochondria and peroxisomes to TG, which is involved in VLDL formation [53]. The high VLDL, HDL, and GLU levels obtained may be caused by the monitoring role of GLU on their excretion from the liver and blood.

## Conclusion

This study showed that parturition and the first 12 weeks of lactation are critical physiological stages because most metabolic changes occur during this period. Therefore, through our results, circulating hormones and metabolic parameters will help breeders and veterinarians for the best management of reproduction to improve production in indigenous Saharan goats. Furthermore, PRL, CORT, and metabolite parameter levels can also serve for early diagnosis and prognosis of blood changes due to metabolic disorders during these physiological stages. Moreover, even though the udder of this breed had a small size, a suspensory system of medium strength, and teats of a shape not entirely favorable to milking, it revealed perfect endocrine and metabolic profiles that maintained lactation and ensured the growth of its offspring under the hostile conditions of its biotope. Thus, we can conclude that this Saharan breed presents adaptive responses expressed by metabolic and hormonal variations modulated by physiological cellular and molecular mechanisms. Hence, it is important to deepen the endocrine and energetic metabolism involvement in this breed, particularly by assessing estradiol 17b, thyroid hormones, NEFA, and b-hydroxybutyrate.

## Authors’ Contributions

KH: Carried out animal experimentation, biochemical assays, analyzed the results, and wrote the article. SB: Carried out the statistical analysis. FK: Coordinated the research activity planning and execution and commented on the manuscript. ZA: Interpretation of the results and English editing. DC: Performed the prolactin assays. SC: Designed the study, interpretation of the results, and drafted and revised the manuscript. All authors read and approved the final manuscript.
